# Complexation with Alginate in Pumpkin Leaf Protein Solutions for the Encapsulation of Folic Acid: The Effect of Extraction Protocols

**DOI:** 10.3390/foods13223695

**Published:** 2024-11-20

**Authors:** Predrag Petrović, Bojana Balanč, Jelena Mijalković, Tamara Đukić, Stefan Bošković, Verica Đorđević, Branko Bugarski, Viktor Nedović, Zorica Knežević-Jugović

**Affiliations:** 1 Innovation Centre of Faculty of Technology and Metallurgy, University of Belgrade, Karnegijeva 4, 11000 Belgrade, Serbiatdjukic@tmf.bg.ac.rs (T.Đ.); sboskovic@tmf.bg.ac.rs (S.B.); 2 Faculty of Technology and Metallurgy, University of Belgrade, Karnegijeva 4, 11000 Belgrade, Serbia; jjovanovic@tmf.bg.ac.rs (J.M.);; 3 Faculty of Agriculture, University of Belgrade, Nemanjina 6, 11080 Beograd, Serbia

**Keywords:** complexation, pumpkin leaves, protein, RuBisCO, folic acid, alginate

## Abstract

This study aimed to assess pumpkin leaves as a protein source and determine the feasibility of these proteins to form complexes with alginate for the encapsulation of folic acid. Different isolation protocols, two based on isoelectric precipitation (one with thermal pretreatment and the other with alkali pre-extraction) and one based on stepwise precipitation with ammonium sulfate, were compared regarding the yield and structural properties of the obtained leaf protein concentrates (LPC). The highest purity of protein was achieved using the thermal-acid protocol and the salting-out protocol at 40% saturation. RuBisCO protein was detected by SDS-PAGE in all LPCs, except for the fractions obtained through salting-out at saturation level ≥ 60%. Complexation of the LPC solutions (1 mg/mL) and sodium alginate solution (10 mg/mL) was monitored as a function of LPC:alginate ratio (2:1, 5:1, and 10:1) and pH (2–8) by zeta-potential measurements and confirmed by FT-IR analysis. Based on the results, the strongest interaction between LPCs and alginate occurred at a pH between 2.20 and 2.80 and an LPC:alginate ratio of 10:1. Complexation resulted in particle yields of 42–71% and folic acid entrapment of 46–92%. The LPC-folic acid interactions elucidated by computational protein–ligand docking demonstrated the high potential of RuBisCO as a biocarrier material for folic acid. The in vitro release study in the simulated gastrointestinal fluids indicated that complexes would be stable in gastric conditions, while folic acid would be gradually released in the intestinal fluids.

## 1. Introduction

Proteins and charged polysaccharides can form different structures in solutions due to associative electrostatic interactions. Depending on their physicochemical properties and concentration, as well as external factors (ionic strength, pH), proteins and polysaccharides may form coacervates, complexes, and gels [1,2]. Coacervates are created when a liquid-liquid phase separation (coacervation) occurs in a colloid solution and represent the phase in which colloids are concentrated [2]; they appear as spherical aggregates that remain in a liquid state, having emulsion-like characteristics. Protein–polysaccharide interactions, however, more often lead to the formation of complexes, which, depending on conditions, may remain soluble or may phase separate as solid co-precipitates. Complexes are sometimes referred to as coacervates in the literature, and vice versa, though they have different properties [1]. Biopolymer complexes and coacervates have a high possibility for application in the food industry as they can have a wide range of functionalities in various food products. They are used for encapsulation, biomimetic systems, packaging films, and as food emulsions or gels [1,2,3]. Many animal-derived (milk proteins and gelatin) and plant-derived proteins extracted from various sources such as legumes (soybean, pea, and bean), cereals (rice, wheat, and maize), and seeds (canola, chia, rape seed, flaxseed, and hemp seed) have been used for coacervate structuring [3,4,5,6].

With the growing world population, there is a need to shift to cheap protein sources. Green leaves of agricultural plants such as pumpkin, duckweed, sunflower, sugar beet, spinach, and alfalfa have the potential to be a sustainable and abundant source of protein [7,8]. Huge quantities of green leaves from certain crops remain on fields as waste or are discarded in some other way after the cultivation cycle. The main green leaf protein constituent is RuBisCO, one of the most abundant enzymes and proteins on earth, which plays an important role in the first major step of carbon fixation, i.e., the conversion of carbon dioxide from the atmosphere into glucose and other energy-rich molecules [9]. RuBisCO consists of two types of protein subunits, called the large chain (55 kDa) and the small chain (13 kDa). Some of its functional properties have been investigated, such as its emulsifying properties [10,11] and its ability to form foams and gels [7,10]. However, the behavior of leaf proteins in solution regarding the formation of self-assembled particles and their capacity to form coacervates by interacting with polysaccharides has been barely considered.

Usually, the isolation procedures for the recovery of leaf proteins contain the following steps: first, mechanical plant cell disruption; second, precipitation by heat, pH, or a combination of both; and third, protein concentration [12,13]. The fractionation process affects not only protein solubility and functionality but also the ability to form coacervates, as recently showed on pea protein by Kornet et al. [14]. In fact, molecular and macromolecular crowders, which are present as impurities in protein fractions, can significantly influence protein–protein interactions. They can also lead to markedly different compositions and structures of the complexes formed in their presence [15]. The isolation steps should eliminate the impurities (fibers, pigments, phenolics, and antinutritional factors) from the mixture to reach the leaf protein concentrate. However, a purer protein fraction does not necessarily mean a better protein fraction in terms of functional properties. A balance should be found between the removal of unwanted compounds and damaging the protein on one side and between the quality of the protein fraction (in terms of technical functionality) and the costs of purification on the other side.

Alginate is a polysaccharide that carries a net negative charge in a wide pH range. It showed the ability for the formation of coacervates with a number of commonly used proteins (guar gum, collagen, gelatin, and pea protein) and also with newly emerged proteins such as a protein isolate from an insect, cricket [16]. To extract protein from pumpkin leaves, three different isolation protocols were used in this study. The two are common methods; one is based on precipitation by heat and acid, and the other on alkali solubilization and acid precipitation. The third is an uncommon isolation protocol used so far to fractionate membrane proteins, based on salting-out precipitation. Salting-out is low-cost, environmentally friendly, and does not cause the denaturation of proteins. This method is based on the effect of high salt concentrations on decreasing protein solubility, which leads to precipitation [17]. Since proteins precipitate at a specific concentration of salt, this procedure has the advantage that the proteins of interest can be separated from other proteins and precipitated. Tola and Misshoun [18] have recently fractionated the total protein extract obtained from *Arabidopsis thaliana* leaf through three consecutive saturation levels of ammonium sulfate (40%, 60%, and 80%) and gained fractions of different electrophoretic profiles.

With this in mind, the objective of the current study was to examine the effectiveness of leaf protein isolated at a laboratory scale from pumpkin (*Cucurbita pepo*) by different fractionation protocols for forming complexes with alginate. Specifically, the aim was to determine which of the fractions exhibits the best performance in terms of forming complexes with alginate, which can serve as vitamin carriers. Folic acid (vitamin B9) is selected as a model vitamin. It is an essential vitamin, meaning that it is not synthesized by the body and must be supplied by diet or other external sources. There is a need to develop encapsulated forms for improvement of its bioavailability as it is poorly soluble in water across a broad range of pH levels (10 mg·L^−1^ at pH 7) and highly sensitive to harsh environmental conditions such as heat, acid environment, and oxidants. So far, folic acid has been encapsulated in complex coacervates based on whey proteins [19,20,21,22].

## 2. Materials and Methods

### 2.1. Plant Materials and Chemical Reagents

The green leaves of pumpkins were collected from 18-week-old soil-grown plants at harvest time (JS&O d.o.o Novo Miloševo, Novo Miloševo, Serbia). The collected leaves were cleaned and stored in a deep freezer at −80 °C before being used for protein isolation.

The chemical reagents that were required for spectrometric analysis of polyphenols, carbohydrates, and protein content: gallic acid, glucose, and Folin and Ciocalteu’s phenol reagent were purchased from Sigma Aldrich Co. (St. Louis, MO, USA). All other chemical reagents used were of analytical grade. The deionized water used to prepare samples (18.2 M MΩ.cm) was generated using a Milli-Q purification system (Merck Millipore Advantage A10, Darmstadt, Germany).

### 2.2. Leaf Protein Concentrate Extraction Procedures

**Figure 1:**
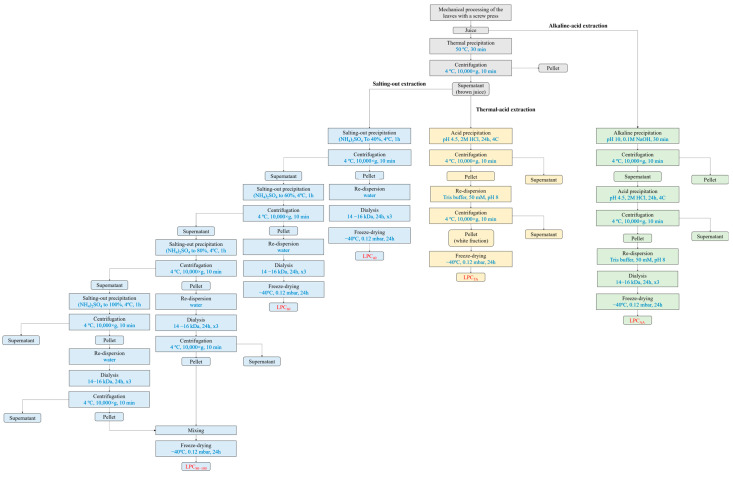
Schematic overview of the fractionation processes used to yield LPC_TA_ (leaf protein concentrate obtained after thermal-acid extraction), LPC_AA_ (leaf protein concentrate obtained after alkaline-acid extraction), and LPC_40_, LPC_60_ and LPC_80–100_ obtained by stepwise precipitation with ammonium sulfate to 40%, 60%, and 80–100% saturation, respectively.

The fractionation steps of the three isolation methods applied in this study are visualized in Figure 1. In all three protocols of protein isolation, the first step is mechanical treatment to disrupt the cells and allow proteins to be solubilized. The pumpkin leaves were pressed with an Angel-8500s slow juicer (Angel, Naarden, The Netherlands), separating a crude juice fraction and a solids fraction. The juice fraction was used for the production of protein concentrate products upon removal of impurities and solid residues by centrifugation at 3000× *g*, 10 min at 4 °C (Optima XPN-100 Ultracentrifuge, Beckman Coulter, Inc., Brea, CA, USA).

### 2.3. Chemical Composition of Leaf Protein Concentrate

In the thermal-acid extraction process, the crude juice was heated to 50 °C and kept at this temperature for 30 min to denature and precipitate the “green fraction”, consisting mostly of chloroplasts. Afterward, the juice was centrifuged at 10,000× *g* for 10 min (Optima XPN-100 Ultracentrifuge, Beckman Coulter, Inc., Brea, CA, USA) to collect the supernatant. The pH of the supernatant (brown juice) was adjusted to 4.5 with 1 M HCl, and the precipitated proteins were separated by centrifugation (10,000× *g*, 10 min, 4 °C). The precipitate, now present in the pellet, was washed with a small volume of Tris buffer (50 mM, pH 8), centrifuged (10,000× *g*, 10 min, 4 °C), and the collected pellet was freeze-dried (−40 °C, 0.012 mbar, 24 h) to obtain leaf protein concentrate labeled as LPC_TA_.

In the alkaline-acid extraction protocol, the pH of the crude juice was first adjusted to 10. After a 30 min incubation at room temperature, the juice was centrifuged (10,000× *g*, 10 min, 20 °C), and the precipitate, containing the green fraction, was discarded. The supernatant was adjusted to pH 4.5 using 1 M HCl solution. After incubation for 30 min with stirring and centrifugation (10,000× *g*, 10 min, 4 °C), a precipitate containing the white protein fraction was collected. The precipitate was resuspended in Tris buffer (10 mM, pH 8) and dialyzed for 24 h against deionized water in cellulose–acetate dialysis tubes (Servapor MWCO 14–16 kDa, Serva, Electrophoresis GmbH, Heidelberg, Germany). The dialyzed sample was collected and freeze-dried under the same conditions as in the previous protocol. In this way, LPC_AA_ was obtained.

In the third method of protein isolation, the green fraction was removed as described in the thermal-acid extraction protocol. The supernatant (brown juice) was then subjected to protein precipitation using ammonium sulfate. Proteins were fractionated based on their differential solubility at 40, 60 and 80–100% saturation in ammonium sulfate, resulting in three fractions labeled LPC_40_, LPC_60_, and LPC_80–100_. Specifically, brown juice was combined with a calculated mass of ammonium sulfate to achieve 40% saturation, with constant stirring using a magnetic stirrer (700 rpm; IKA RCT basic, IKA-Werke GmbH & Co. KG, Breisgau, Germany) at room temperature for 15 min. The precipitated protein was collected by centrifugation (10,000× *g* for 10 min at 4 °C), and the resulting pellet was designated as LPC_40_. The supernatant was then subjected to higher ammonium sulfate saturation (60–100%). The pellets obtained for 40%, 60%, and 80–100% were dissolved in a minimal volume of water. All three fractions were subjected to dialysis in cellulose–acetate dialysis tubes (Servapor MWCO 14–16 kDa, Serva, Electrophoresis GmbH, Heidelberg, Germany) against deionized water for 24 h to remove excess ammonium sulfate and other small compounds. The tube contents were then collected and freeze-dried.

The LPC samples were chemically characterized by analyzing their protein and carbohydrate content, as well as the antioxidant capacity (“polyphenol content”).

Protein content was determined using the Kjeldahl method [23] on an Exclusive Kjeldahl workstation (behr Labor-Technik, Düsseldorf, Germany); the elemental nitrogen content of the LPC sample was determined and converted to protein content using a literature conversion factor of *n* × 6.25.

The phenol–sulfuric acid method was used to determine the amount of carbohydrates present in the LPC samples [24]. To prepare a known sample concentration for each sample, the dry LPC was reconstituted in deionized water (1 mg/mL). 2 mL of concentrated sulfuric acid and 0.4 mL of phenol (5%, *w*/*v*) were combined with aliquots of 0.4 mL sample solution (0.5 mg/mL). The mixtures underwent incubation at 25 °C for 40 min. A spectrophotometer (Shimadzu UV-1800, Kyoto, Japan) was then utilized to measure the absorbance at 490 nm against a blank sample that was made with water instead of the LPC sample solutions. Following a comparison of the absorbance data with a glucose standard curve, the results were represented as a percentage. All measurements were performed in triplicate.

With minor adjustments, the Folin–Ciocalteu method was used to analyze the total phenolic content of the LPC samples [25]. Aliquots of a 0.1 mL sample (10 mg/mL), 6 mL of deionized water, and 0.5 mL of Folin–Ciocalteu reagent were added to 10 mL test tubes and thoroughly mixed. After incubating for three minutes at 25 °C, 1.5 mL of 20 wt.% Na_2_CO_3_ was added to the tubes, after which mixtures were adjusted to 10 mL with water. The mixtures were kept in the dark at 25 °C for 2 h, and afterward, absorbance was measured at 630 nm (Shimadzu UV-1800, Kyoto, Japan). Gallic acid was used as a standard (0–1 mg/mL), and the results were expressed as mg of gallic acid equivalents per gram of LPC powder. All measurements were performed in triplicate.

### 2.4. Analysis of Protein Purity and Subunit Molecular Weights

Sodium dodecyl sulfate–polyacrylamide gel electrophoresis (SDS-PAGE) was used to determine the profile and molecular weight of proteins present in LPCs in accordance with Laemmli’s technique [26]. The LPC stock samples were combined with sample buffer, consisting of the following ingredients: 2.5 mL of 2X Tris-HCl buffer, 2.0 mL of glycerol, 2.0 mL of 1 M DTT solution, 4.0 mL of 10% (*w*/*v*) sodium dodecyl sulfate, and 0.4 mL of 5% (*w*/*v*) bromophenol blue, at a volume ratio of 1:1. The mixture was then heated for five minutes at 95 °C. After cooling and without waiting, the reduced and denaturated LPC samples were loaded onto a polyacrylamide gel (mPAGE™ 12% Bis-Tris Precast Gel; 10 × 8 cm, 12-wells; Merck KGaA, Darmstadt, Germany) together with prestained protein ladder standard markers (10–260 kDa; Spectra Multicolor Broad Range Protein Ladder; Thermo Scientific, Waltham, MA, USA). Electrophoresis protein migration was performed using a commercial Tris-MOPS SDS running buffer (Merck KGaA, Darmstadt, Germany) under 120 V for a 70 min run time in a vertical mini-Hoefer system (Hoefer, Holliston, MA, USA). The gels were stained with Coomassie brilliant blue R-250, after which the gels were decolorized over two hours employing an acetic acid/methanol/water (1:4.5:4.5, *v*/*v*/*v*) solution with a few changes.

### 2.5. Preparation of LPC-Alginate Coacervates

To investigate the formation of LPC-alginate complexes as affected by the mixing ratio R (=LPC: alginate, *w*/*w*) and the pH, appropriate volumes of biopolymer stock solutions (1 mg/mL of LPC and 10 mg/mL of sodium alginate) were mixed. The mixing was performed under magnetic stirring at room temperature for 1 h to obtain the following R (*w*/*w*): 2:1, 5:1, and 10:1. The pH was adjusted to each mixture’s isoelectric point by adding HCl. The isoelectric points were determined by measuring the ζ-potential (ζ, mV) of each mixture using a Zetasizer Nano Series Nano ZS (Malvern Instruments, Malvern, UK) in a pH range from 8.0 to 2.0. A dispersed phase refractive index of 1.59 was used at 25 °C.

### 2.6. FTIR

The IR spectra of complexes were recorded in the transmission mode between 400 and 3700 cm^−1^ using a Nicolet iS10 FTIR spectrometer (Thermo Fisher Scientific, Waltham, MA, USA), operating in ATR mode.

### 2.7. Light Microscopy

Light microscopy images were captured using a Motic light microscope (BA 210, Xiamen, China) with a Moticam digital camera (1 SP, 1.3 MP). Images were processed in Motic Images Plus 2.0 software.

### 2.8. Complex Coacervate Yield (Cy)

The yield of the LPI-alginate complex coacervation process was measured by collecting (after centrifugation at 4000× *g* for 10 min) and freeze-drying the formed complexes. The following equation was used:

$$C_{y}=\frac{m}{m_{LPC}+m_{alginate}}\times 100\%$$

where m is the weight of freeze-dried complexes and m_LPC_ and m_alginate_ the masses of LPC and alginate, respectively, used to prepare the corresponding LPC-alginate suspensions. All experiments were performed in triplicate.

### 2.9. Encapsulation of Folic Acid and Encapsulation Efficiency (EE)

The stock solution of folic acid (5 mg/mL) was prepared in water by adjusting pH to 9 with 0.1 M NaOH. The solution was then mixed with the LPC solutions (1 mg/mL, pH set to 8) so that the LPC and folic acid weights were in a 10:1 ratio. After a short mixing, the alginate solution was added dropwise into the mixture to make different LPC:alginate ratios. The pH of the solution was adjusted to the previously determined isoelectric points. The final mixture was centrifuged (7000× *g*, 12 min), and the precipitates were collected and lyophilized. The supernatant was further used to indirectly determine folic acid content in sedimented particles. The supernatants were filtered using a 0.2 µm filter (MACHEREY-NAGEL, Düren, Germany), and the absorbance of the filtrates was measured at 365 nm (Shimadzu UV-1800, Kyoto, Japan). The content of folic acid in coacervates was calculated as the difference between the initial concentration of folic acid (used for the preparation of coacervates) and the concentration detected in the supernatant. A standard curve of folic acid was constructed in the range of concentrations from 0.039 to 0.125 mg/mL.

### 2.10. Molecular Docking

CB-Dock 2 software [27,28], which can be accessed from https://cadd.labshare.cn/cb-dock2/index.php (accessed: 17 June 2024), was used for molecular docking simulations. Only the single best result (according to Vina score) was used in the analyses. The molecule structures acquired from UniProt [29] were ribulose bisphosphate carboxylase large chain (ID: P48697); ribulose bisphosphate carboxylase small subunit, chloroplastic (ID: A0A6J1G227); ribulose bisphosphate carboxylase large chain (ID: P00875); ribulose bisphosphate carboxylase small subunit, chloroplastic 2 (ID: Q43832); whey acidic protein (ID: O46655); glycinin G2 (ID: P04405); PubChem [30] (sodium alginate (CID: 5102882); and folic acid (CID: 135398658).

### 2.11. Scanning Electron Microscopy

The surface morphology of the selected LPC:alginate complex, as well as the complex with folic acid, was analyzed by scanning electron microscopy (SEM, model TESCAN MIRA3XMU, Brno, Czech Republic) using a voltage of 10 kV and a magnification of 10,000. Previously, the samples were coated with gold under vacuum.

### 2.12. Release Kinetics of Folic Acid in Simulated Gastrointestinal Medium

In vitro gastrointestinal (GI) digestion catalyzed by pepsin and pancreatin was performed in a batch system by the described method from Liu et al. [31]. The controlled release was monitored in two types of solutions that simulate the GI conditions: gastric and pancreatic fluid.

Simulated gastric fluid (SGF) was made by dissolving 0.5 g NaCl in 1.25 mL of a 6 M HCl solution. The solution was diluted with 200 mL of distilled water, the pH was corrected to 1.4–1.5, and the volume was filled to 250 mL. Just before use, the starting solution was incubated at 37 °C for 30 min. The SGF was finally made by dissolving the pepsin enzyme (3.2 mg/mL) in the heated starting solution. The pH was set to 1.4.

Simulated intestinal fluid (SIF) was prepared by dissolving 1.7 g of dipotassium hydrogen phosphate in 47.5 mL of 0.1 M sodium hydroxide, and pH was adjusted to 7.4. Bile salts at a concentration of 0.2 mg/mL were added, and the flask was filled up to 250 mL with water. This prepared stock solution was incubated for 30 min at 37 °C with constant stirring. The pancreatin solution was prepared immediately before use by dissolving pancreatin (3.2 mg/mL) in the heated stock solution. After dissolution, the pH was adjusted to 7.4.

The selected LPC:alginate complex, as well as the complex with folic acid, was suspended in SGF/SIF at a concentration of 2 mg/mL and incubated at 37 °C in an orbital shaker (IKA KS 3000, Staufen, Germany) with gentle shaking. Samples were collected from the release medium at predetermined time intervals. Folic acid release was quantified by measuring the absorbance at 361 nm, up to the point where the maximum release was reached and stabilized. Absorbance values were recorded as the difference between the sample containing folic acid and its counterpart without folic acid, measured at the same time point and wavelength. Release measurements were performed in triplicate.

### 2.13. Statistical Analysis

All experiments were performed in duplicate or triplicate, and the results are expressed as mean ± standard deviation. The results were statistically analyzed using one-way analysis of variance ANOVA (Origin software OriginLab Corporation, Northampton, MA, USA). The *t*-test was used to compare mean differences between the samples. The significance level was 0.05.

## 3. Results and Discussion

### 3.1. Comparison of the LPC Fractions

**Figure 2:**
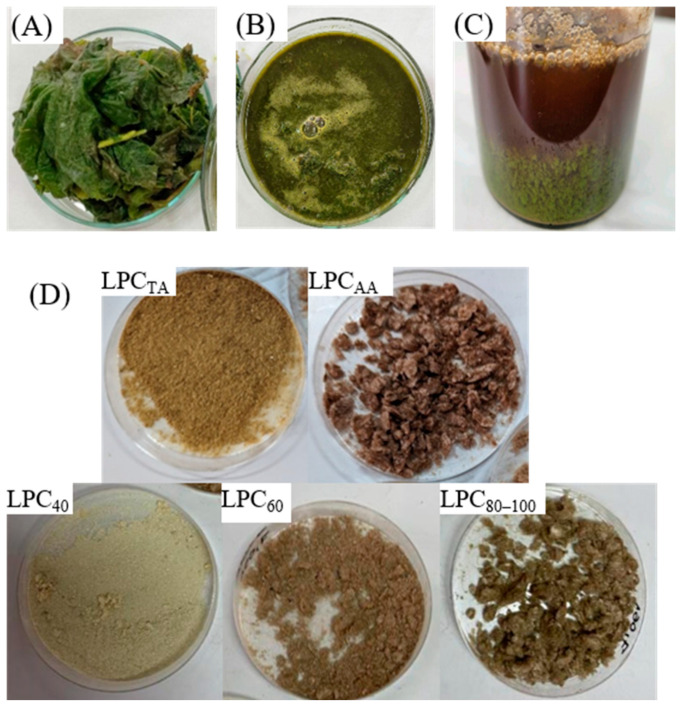
Raw pumpkin green leaves (**A**); green juice obtained after screw pressing step (**B**); brown juice (**C**) and leaf protein concentrate powders obtained by different extraction protocols (**D**).

The extraction protocol comprised mechanical processing of the green leaves (Figure 2A) to obtain crude juice (Figure 2B), followed by a heating step at relatively mild temperature (in case of thermal-acid and salting-out protocols) to achieve aggregation of insoluble proteins and cell debris containing chlorophyll (“green fraction”), while soluble proteins remained in the supernatant (“white protein fraction”) (Figure 2C). In the salting-out protocol, stepwise precipitation with ammonium sulfate at saturations of 40%, 60%, 80%, and 100% produced four fractions of leaf protein concentrate, designated LPC_40_, LPC_60_, LPC_80_, and LPC_100_. LPC_40_ yielded the highest total recovered biomass at 56.1%, followed by LPC_60_ at 28.1%, LPC_80_ at 9.3%, and LPC_100_ at 6.5%. Due to the very low yields of LPC_80_ and LPC_100_, as well as their similar physicochemical properties (such as color, solubility, and FTIR spectra), these two fractions were combined into a single fraction (LPC_80–100_) for coacervation purposes.

The fractions differed in terms of color and solubility. The color changed from off-white in the case of LPC_40_ to light brown for LPC_60_, light yellow-brown for LPC_TA_, and dark brown for LPC_80–100_ and LPC_AA_. The yellowish/greenish tint of some of the samples is a consequence of the fact that heat precipitation is not highly selective, and in practice, both cytoplasmic and chloroplast proteins can be found in both soluble and non-soluble fractions [12,32]. Furthermore, the presence of the phenolic compounds and the enzyme polyphenol oxidase may lead to a browning reaction and the formation of melanin pigments, which causes a darker coloration in some samples. The results obtained from protein extraction by salting-out protocol indicated that the increase in the ionic strength of the solvent resulted in polyphenol oxidase precipitating at higher ionic strengths since the browning of the samples increased with ammonium sulfate saturation level.

According to the results shown in Table 1, the protein content was between 63 and 90% and changed in the order LPC_40_ > LPC_TA_ > LPC_AA_ > LPC_60_ > LPC_80–100_. In addition, the results of the carbohydrate content determined by the phenol-sulfuric acid method and the total phenolic content measured by the Folin–Ciocalteu assay are presented in Table 1. In the case of samples obtained by the salting-out protocol, the yields of protein in fractions decreased with increasing salt concentration, while the carbohydrate content was in the complementary sequence of the protein content (LPC_40_ < LPC_60_ < LPC_80–100_). The increase in carbohydrate content coincided with better solubility, indicating the possible formation of soluble complexes between proteins and carbohydrates. The thermal-acid extraction protocol resulted in the lowest carbohydrate content (below 4%).

The highest total phenolic content (TPC) was determined for LPC_60_, and the lowest for LPC_40_. The phenolic groups in proteins originate from the tyrosine side chain, although tryptophan and cysteine also display significant reactivity towards the Folin–Ciocalteu reagent, as well as many other non-phenolic compounds with pronounced antioxidant/reducing ability [33]. Besides proteins, pigments, such as melanins (resulting from browning reactions), may also possess phenolic moieties in their structure [34], which was evident by the higher TPC of pigmented samples (LPC_AA_, LPC_60_, and LPC_80–100_, compared to LPC_40_). Free phenolic compounds might contribute to the relatively high TPC of LPC_TA_, which was the only sample obtained without dialysis.

The total crude protein concentrate yield based on dry matter was 0.92% in alkaline-acid, 1.31% in thermal-acid, and 3.94% in salting-out extraction. In general, studies evaluating “white protein” extracts from green biomass report different yields even from the same crop type. There are several reasons: (1) the protein content depends on the plant source (variety, cultivation conditions, and harvest stage); (2) extraction procedures largely differ between studies; and (3) yield calculations combine different methods and fractions in different reports. Recently, Perović et al. [35] recovered a white protein fraction from pumpkin leaves at 2.7% (measured by Bradford assay) by enzyme-assisted protocol. Ghaly and Alkoaik [36] reported the highest protein yield for pumpkin leaves (11.75% dry basis) out of the six tested crops grown in Africa; the concentrate was obtained by an alkaline-thermal extraction protocol combined with intensive two-step mechanical processing (blending and pressing).

**Figure 3:**
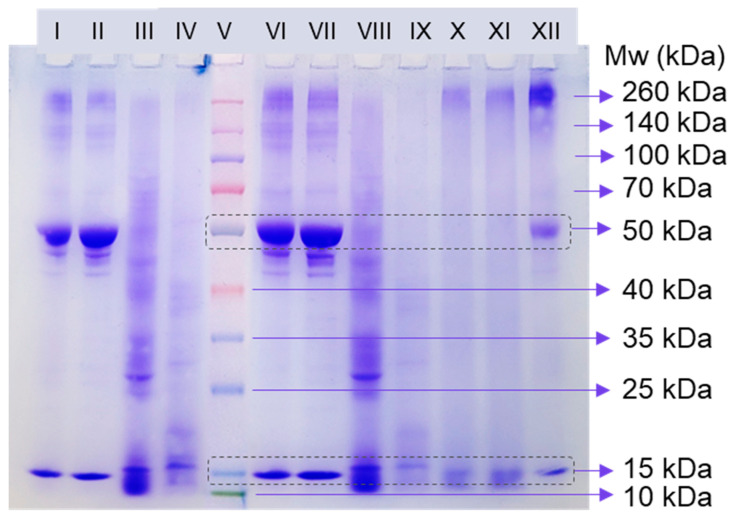
Electrophoretic profiles of the LPCs isolated via various extraction protocols from pumpkin leaves; bands: I and VI—LPC_TA_; II and VII—LPC_40_; III and VIII—LPC_60_; IV and IX—LPC_80–100_; V—multicolor protein ladder; X and XI—LPC_AA_ prepared with reduced sample buffer; and XII—LPC_AA_ prepared with non-reduced sample buffer.

SDS-PAGE was used to examine the protein profile in the LPC fractions and to determine RuBisCO in order to verify its existence and purity. Native RuBisCO is a multimeric protein made up of eight (~15 kDa) and eight large subunits (~50 kDa), as shown by quaternary structural studies [8]. The comparison of the electrophoretic profiles with molecular weight protein markers demonstrated the good visibility of the two RuBisCO subunits, as seen in the electrophoretic profiles of LPC_TA_ and LPC_40_ under reducing conditions and LPC_AA_ under non-reducing conditions (Figure 3). Additionally, some minor bands were visible immediately below the RuBisCO’s 50 kDa band; these bands appear to be the consequence of slight protein degradation that occurred during separation as a result of leftover proteases [37]. The protein subunit profiles of the LPC_40_ and LPC_TA_ samples (bands I–VI and II–VII) matched completely, revealing that acidic precipitation and precipitation with 40% ammonium sulfate yielded identical protein fractions with comparable subunits. The proteins produced from both of these procedures were highly pure, with the largest proportion of RuBisCO proteins. Nonetheless, protein bands over 100 kDa were also detected, which are commonly associated with polyphenol oxidase, carbonic anhydrase, phosphoenolpyruvate carboxylase, and other enzymes that typically occur in the C3-C4 plant cell of the leaf [38]. Furthermore, this may be a result of the protein fragments in LPC_TA_ and LPC_40_ being hydrophobic, which causes a high degree of polymerization and is manifested as a band of proteins with a molecular weight greater than 100 kDa [39].

In contrast, the electrophoretic profile of the LPC_AA_ sample is primarily characterized by strongly stained diffuse bands (data not provided). So, non-reducing conditions were used (Figure 3, band XII), and the RuBisCO subunits are visible, as well as the protein fragment mass of ≥260 kDa. The protein lines are, however, blurry, likely owing to the proteins’ poor solubility in the sample buffer. The SDS-PAGE of the LPC_60_ and LPC_80–100_ did not reveal pure protein fragments, and the profiles do not exhibit characteristic bands of RuBisCO subunits, suggesting that precipitation with salts of high concentration (60, 80, and 100% *w*/*w*) is unfavorable for the isolation of hydrophobic proteins as RuBisCO is. Instead, bands in the low molecular range were observed (>15 kDa), especially in LPC_80–100_, which exhibited high water solubility.

Overall, based on the protein band profiles, we may infer that all three protein separation procedures are appropriate for obtaining a protein concentrate with a relatively high content of the RuBisCO subunits, and the thermal-acid and salting-out protocol via 40% ammonium sulfate fraction would be the best choices.

**Figure 4:**
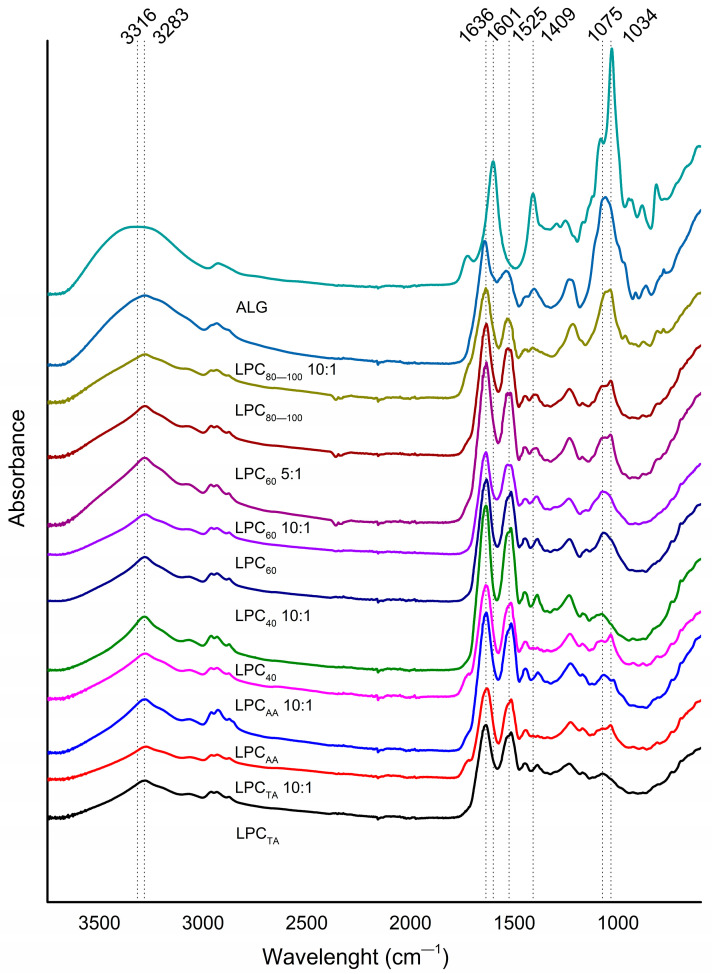
FTIR spectra of LPC fractions, LPC-alginate complexes (prepared at different mixing ratio) and sodium alginate (ALG).

In the FTIR spectra (Figure 4), all the LPC samples showed the same distinctive bands, but their intensity differed among samples. Amide I (1636 cm^−1^) and Amide II band (1525 cm^−1^), originating from peptide structures, were dominant bands in all samples, confirming the protein nature of the samples. Amide I correlates with the C=O stretching, while the Amide II band arises from N–H bending. The Amide III band was expected at 1300–1200 cm^−1^, but it usually overlaps with other non-specific bands. Also, there was a relatively broad band in the range between 3600 and 3000 cm^−1^ (peaking at 3283 cm^−1^), arising from secondary amine N-H stretching vibrations, and is associated with the peptide backbone. This band often overlaps with the band originating from stretching vibrations of the hydroxyl groups (O-H), which is broader without a well-defined peak. The presence of both bands, however, is pronounced in the LPC_80–100_ spectrum and hints at the highest carbohydrate content of this fraction, since carbohydrates are rich in OH groups. Multiple overlapping bands between 3000 cm^−1^ and 2800 cm^−1^ correspond to C-H stretching of CH, CH_2_, and CH_3_ groups, associated with the presence of proteins and also carbohydrates. The most prominent difference among samples can be seen in the so-called “sugar region”, between 1200 cm^−1^ and 800 cm^−1^, consisting of multiple bands (with the most pronounced peak being at ~1075 cm^−1^) that correspond to C-O stretching and C-O-H bending vibration, typical of carbohydrate structures. In this region, LPC_80–100_ gave the bands of the highest relative intensity, followed by LPC_60_ and then the rest of the samples (LPC_40_, LPC_AA_, and LPC_TA_). The intensity of the Amide I and II bands (relative to bands of the sugar region) was higher in LPC_40_, LPC_AA_, and LPC_TA_ than that of the other two salting-out fractions. This result corresponds to the protein and carbohydrate contents of the extracts determined in chemical analysis.

### 3.2. Complexation Between Alginate and Leaf Protein as a Function of pH

The LPC solutions (1 mg/mL) and sodium alginate solution (10 mg/mL) were characterized in terms of ζ-potential as a function of pH (2–8). LPCs exhibited neutral ζ-potential values between 2.8 and 4.3, depending on the specific fraction, with the highest isoelectric point for LPC_TA_ and the lowest for LPC_80–100_, which may be due to differences in amino acid composition (ratio of acidic and alkaline amino acids), tertiary and quaternary structure of proteins, and the presence of acidic sugars in the carbohydrate fraction of the samples. The isoelectric point of pure RuBisCO was determined to be between 4.4 and 5.5 in the previous studies, with the exact value being species-specific [40]. Alginate, as an acidic polysaccharide, was negatively charged in the whole pH range. ζ-potential of LPCs at neutral pH was negative (between −20 and −35 mV), and it increased gradually with the decrease in pH. In a highly acidic environment (<pI), the positively charged moieties (−NH_x_^+^) in proteins outnumber the negatively charged moieties (−COO^−^), leading to a net positive surface charge in the protein solution necessary for coacervation with alginate via its negatively charged carboxylic groups.

**Figure 5:**
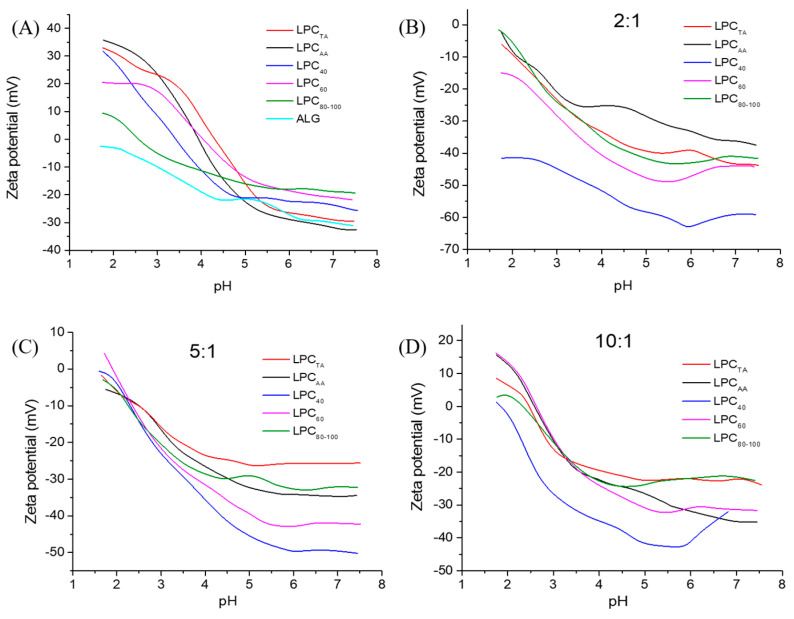
Diagrams of ζ-potential as a function of pH: plain LPC and alginate solutions (**A**), and mixtures of LPC with alginate in ratios of 2:1 (**B**), 5:1 (**C**), and 10:1 (**D**).

The protein–polysaccharide ratio is one of the most important parameters affecting complex formation by altering the charge balance in the complexes. The impact of LPC-alginate ratio was investigated in terms of ζ-potentials and microscopic images. According to ζ-potential values of dispersions (Figure 5), complexes generated at LPC:alginate ratios of 2:1 and 5:1 (in the case of all LPC samples except for the LPC_60_-alginate ratio of 5:1) had a negative ζ-potential in the whole pH range, indicating that the positively charged proteins were inadequate to completely bind negatively charged alginate molecules. At the LPC:alginate ratio of 10:1, the ζ-potential of the dispersions reached positive values at low pH values, indicating an excess of protein. These complexes had a neutral ζ-potential (isoelectric point) between 2.20 and 2.80 depending on the protein fraction, and the isoelectric point of the complexes shifted toward lower pH values compared to the isoelectric points of the LPCs alone. The isoelectric points indicated the presence of a stoichiometric equivalence between protein and alginate charge at the given ratio. According to the literature, complexation is mainly determined by electrostatic attractions, and coacervation tends to be highest when the stoichiometry of the macroion charges is equal to one [2,41]. The yield increased with the increase in LPC protein content (Table 2), and the recovered weight of LPC-alginate coacervates was between 34 and 71%.

**Figure 6:**
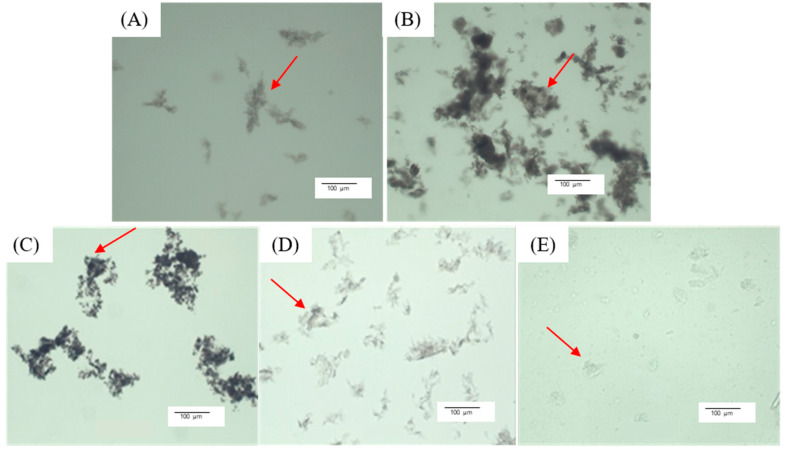
Microscopic images of complexes obtained with LPC_TA_ (**A**), LPC_AA_ (**B**), LPC_40_ (**C**), LPC_60_ (**D**), and LPC_80–100_ (**E**) at LPC:alginate mass ratio of 10:1.

**Figure 7:**
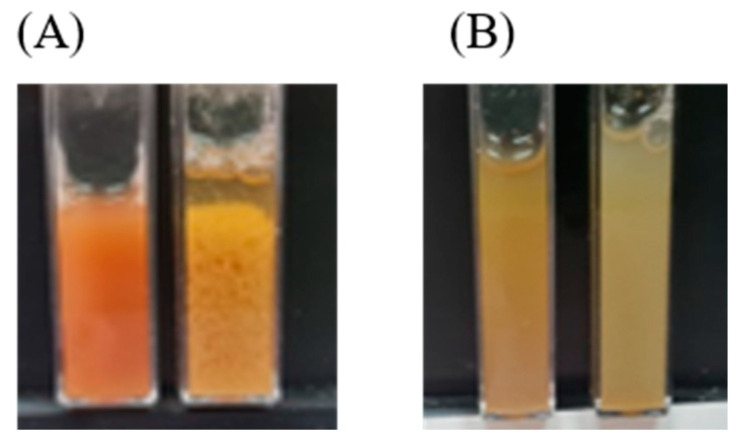
Images of LPC dispersion at pH 8 (left cuvette) and the counterpart complex at isoelectric point (right cuvette) for (**A**) LPC_40_ and (**B**) LPC_80–100._.

Microscopic images (Figure 6) showed complexes of different sizes and densities depending on the LPC fraction used for complexation with alginate at an LPC:alginate ratio of 10:1. The complexes obtained with LPC_40_ represented relatively large, very dense, irregularly shaped particles (>100 μm), so-called ‘‘fractal aggregates’’ described in the previous studies [41,42]. Smaller and less dense particles were obtained with LPC_TA_ and LPC_60_, and the smallest and least dense aggregates with LPC_80–100_. In addition, the co-existence of the aggregates and small irregularly shaped droplets is observed in the LPC_80–100_-alginate sample. These kinds of aggregates are described in the literature as solid precipitates that exhibit amorphous flocculation, while liquid coacervates appear as spherical droplets with a diameter of 1–50 μm [43]. Different phase states of complex condensates were also visually observed; for example, LPC_40_-alginate complexes sedimented faster (Figure 7A), while LPC_80–100_-alginate created a stable colloidal solution (Figure 7B).

The FTIR spectra of alginate and LPC-alginate complexes are shown in Figure 5. Unlike the LPC spectra (analyzed in detail in the previous section), the spectrum of sodium alginate shows characteristic bands of a polysaccharide structure. The broad band between 3600 and 3000 cm^−1^ peaking at 3316 cm^−1^ arises from O-H stretching, from both alcoholic and carboxylic groups. The strong and sharp absorption bands at around 1601 and 1409 cm^−1^ were ascribed to the −COO^−^ asymmetric and symmetric telescopic vibrations, respectively. In addition, there were several overlapping bands of very high intensity in the sugar region (1200–800 cm^−1^), with the absorption peak at a 1034 cm^−1^, arising from C-O-C, −C-H, and −C-O stretching vibrations. The absorption bands of coacervates represented a combination of the bands seen in the LPC and alginate spectrums, with domination by the LPC peptide groups due to the high protein–alginate ratio.

### 3.3. Encapsulation of Folic Acid in Complexes

Theoretically, folic acid can bind to proteins via different functional groups, and it also depends on the protein structure. However, due to its amphoteric nature, the solubility of folic acid in aqueous solutions is strongly pH-dependent and reaches a minimum at a pH range between 2 and 4 (the range at which coacervation occurs) [44]. Due to its low solubility in water, folic acid molecules tend to escape from water into the complexes phase, leading to entrapment within complexes. Similarly, diclofenac sodium soluble in alkaline pH was successively encapsulated in alginate–gelatin coacervates at pH 5.5 [45].

**Figure 8:**
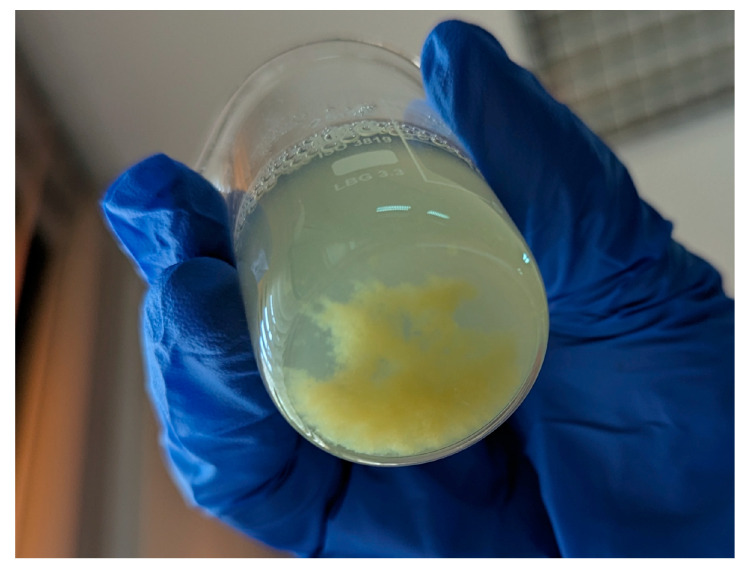
Precipitated complexes of LPC_40_ (LPC:alginate mass ratio of 10:1) showing evenly distributed yellow color of encapsulated folic acid.

At an LPC:alginate ratio of 10:1, the entrapment efficiency varied in a quite wide range, between 46 and 92% depending on the LPC type. For a sake of comparison, complex coacervates composed of β-lactoglobulin and pectin encapsulated folic acid with an efficiency of 58 ± 7% [21]. In our study, the encapsulation efficiency was in correlation with protein content in LPCs, although there were exceptions. Protein type, i.e., molecular weight and structure, also seems to have an effect, such that LPC_60_, although having somewhat higher protein content than LPC_AA_ (80 vs. 75%), exhibited significantly lower entrapment efficiency of coacervates (65 vs. 92%). Namely, according to SDS-PAGE results, LPC_AA_ contained RuBisCO subunits, unlike LPC_60_, which displayed low molecular weight proteins. This means that RuBisCO is a favorable constituent of LPC-coacervates for encapsulation performance (Figure 8). The fraction LPC_40_ had the highest protein content with the RuBisCO as the main constituent; therefore, complexes exhibited the highest encapsulation efficiency. In addition, polyphenols/pigments, as well as carbohydrates present in LPC_60_ and LPC_80–100_ in higher concentrations than in LPC_40_, may saturate/shield some possible binding sites on proteins (Table 2).

### 3.4. Molecular Docking

**Figure 9:**
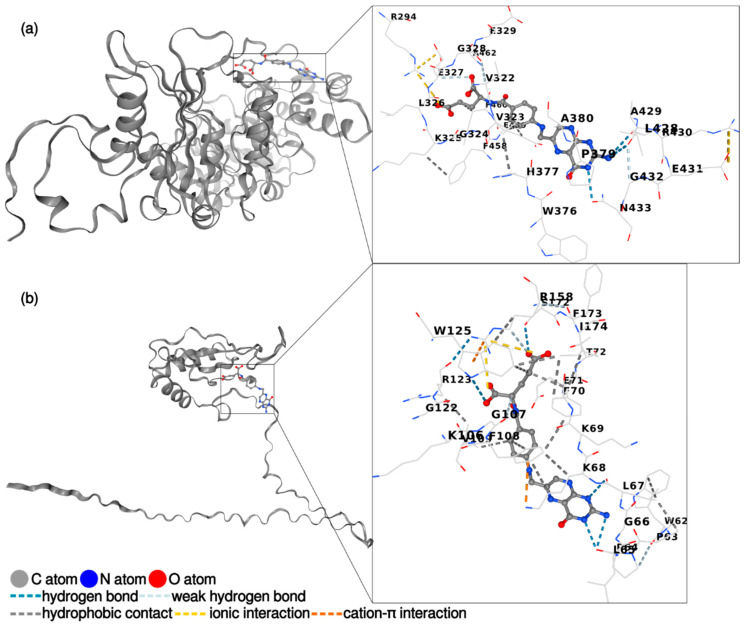
Graphical representation of the interactions between folic acid and (**a**) RuBisCo (large subunit); (**b**) RuBisCO (small subunit).

Molecular docking is used to model the interaction between folic acid (a small molecule) and RuBisCO protein (the main protein in LPC_TA_, LPC_AA_, and LPC_40_) at the atomic level. The CB-Dock 2 software identified the protein residues of RuBisCo from pumpkin involved in binding with folic acid. These residues of the large chain are as follows: ARG294, VAL322, VAL323, GLY324, LYS325, LEU326, GLU327, GLY328, GLU329, TRP376, HIS377, PRO379, ALA380, LEU428, ALA429, ARG430, GLU431, GLY432, ASN433, PHE458, GLU459, PHE460, and ALA462. The molecule of folic acid is composed of three central components: the pteridine ring, para-aminobenzoic acid (PABA), and the glutamate moiety. According to the results depicted in Figure 9a, the pteridine ring forms three hydrogen bonds with the RuBisCo large subunit, *p*-amino-benzoic acid forms two hydrophobic interactions, and glutamic acid forms one weak hydrogen bond, one hydrophobic, and one ionic interaction. The small RuBisCo subunit residues responsible for the interactions with folic acid are TRP62, PRO63, PRO64, LEU65, GLY66, LEU67, LYS68, LYS69, PHE70, GLU71, THR72, LYS106, GLY107, PHE108, VAL109, GLY122, ARG123, TRP125, ARG158, SER172, PHE173, and ILE174 (Figure 9b). The main binding forces are three hydrogen bonds with the pteridine ring, two hydrophobic contacts with para-amino-benzoic acid and two hydrogen bonds, two weak hydrogen bonds, two hydrophobic contacts, and two ionic interactions with the glutamate moiety.

In addition to binding cites, the software predicts the affinity for binding, and this affinity is expressed via the Vine score parameter, which sublimates the contributions of several individual terms. A higher negative Vina score indicates stronger binding affinity. The Vina Score for the folic acid-RuBisCo was equal to −8.3. For the sake of comparison, we computed the interactions between folic acid and other proteins, which have been mostly studied as carriers for folic acid [19,46,47,48,49,50]. The results showed different Vina scores for different proteins: the Vina score was equal to −6.2 for interactions with whey protein (whey acidic protein from *Sus scrofa*), −7.6 for beta-lactoglobulin (from *Bos taurus*), −10.5 for lactotransferrin (human), and −8.2 for glycinin G2-folic acid (from *Glycine max*). This suggests a strong affinity of folic acid toward green leaf protein. There are also other pockets in the RuBisCO structure that could bind folic acid, which are however less favored regarding the binding energy; these pockets could be occupied in the presence of excess folic acid.

### 3.5. Scanning Electron Microscopy

**Figure 10:**
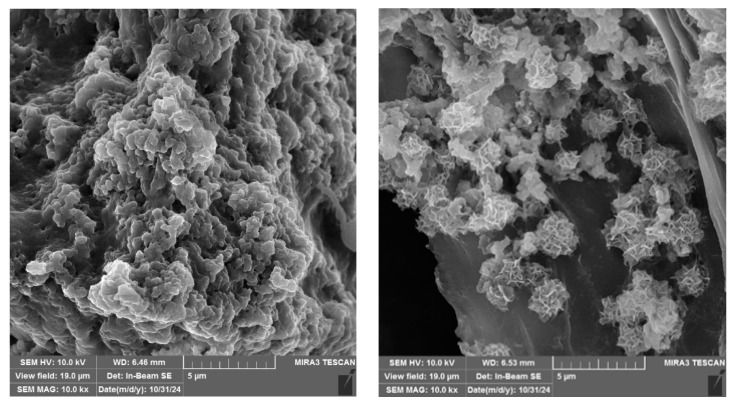
SEM morphologies of LPC_40_:alginate complexes (**left**) and LPC_40_:alginate complexes with encapsulated folic acid (**right**) in 10:1 ratio.

The surface morphologies of LPC_40_:alginate complexes in a 10:1 ratio, both with and without folic acid, were observed using SEM (Figure 10). The LPC_40_:alginate complex exhibited a smooth, spongy granular structure, which can be attributed to the lyophilization process used to remove water. The interaction between alginate and LPC_40_ created a porous network structure. Similar structures were reported by Zhang et al. [51], who studied self-assembled protein–polysaccharide gels under acidic conditions (intragastric). The complexes containing folic acid (Figure 10, right) displayed a porous, flake-like structure that formed clusters. The presence of folic acid seemingly altered these structures, making them more spheroid in shape with pronounced ridges on their surfaces.

### 3.6. Release Kinetics of Folic Acid in Simulated Gastrointestinal Medium

**Figure 11:**
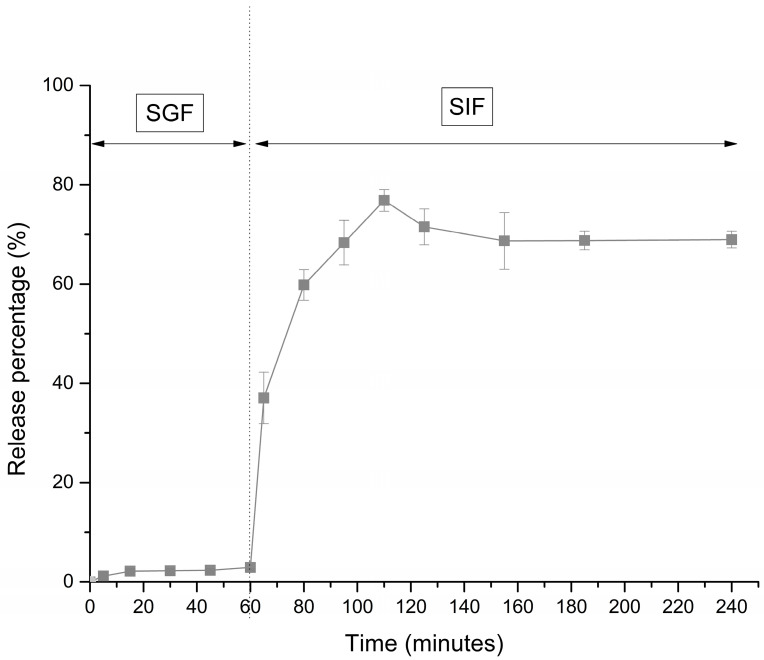
Release profile of folic acid from LPC_40_:alginate complexes in ratio 10:1 in SGF and SIF.

The release study was conducted using complexes prepared with LPC_40_:alginate in a ratio of 10:1 since LPC_40_ was found to be a fraction with the highest protein content and predominantly consisting of RuBisCO. The vitamin was barely released in simulated gastric condition (only 3% after 1 h) and then up to 80% in simulated intestinal medium (Figure 11). Normally, the native structure of protein is expected to be disrupted at low pH and proteolysis (action of pepsin). However, the structure of the complexes seemed to be preserved in the gastric condition, presumedly by alginate molecules. Several studies state that alginates inhibit pepsin activity in vitro via a mechanism whereby ionic interactions reduce substrate availability to the enzyme at acidic pH [52,53]. Another possible explanation is that alginate molecules blocked some of the pepsin target sites at protein molecules. In SGF, the electrostatic interaction between the proteins and alginate was favorable since the biopolymers are charged oppositely at low pH. Also, the complexes created a high viscosity milieu in gastric juice, which retarded diffusion of folic acid and also diffusion of molecules toward and from the enzyme and subsequently slowed down the enzyme–substrate reaction. Similar observations were reported by Wang et al. [54], who showed (based on SDS-PAGE analysis) that the structure of lactoferrin in lactoferrin-sodium alginate complex coacervates was partially protected at the gastric stage of digestion, unlike the unprotected (free) lactoferrin, which was completely broken down to small fragments. In SIF, however, due to a slightly alkaline pH, both alginate and proteins are negatively charged, and the complexes dissociate, making proteins susceptible to pancreatin and proteolysis. Therefore, folic acid was gradually released in SIF, and after 50 min, a concentration equilibrium was observed. According to the literature, folic acid is readily absorbed in duodenum and jejunum [55], thus LPC-alginate complexes would secure the bioavailability of this vitamin.

## 4. Conclusions

Proteins from agricultural waste/biomass can be utilized for the encapsulation of vitamins, which may increase their stability and shelf-life. Leaf proteins may be obtained in different ways, which can alter their physico-chemical properties, i.e., products with pronounced antioxidant activity can be obtained. This study reported the extraction of protein concentrates from green pumpkin leaves by three different isolation protocols: thermal-acid, alkali-acid, and salting-out. The optimum extraction protocol was salting-out, in which a high concentrate yield (2.30%) and the highest protein purity (90%) were achieved upon precipitation with an ammonium sulfate solution to 40% saturation. Almost the entire RuBisCO fraction precipitated at 40% saturation of ammonium sulfate. RuBisCO appeared to be least soluble, or most affected by the changes in the leaf juice microenvironment. The isoelectric point of the LPC samples was between 2.8 and 4.3, depending on the isolation protocol. Despite these differences, all leaf protein concentrates were able to form complexes with alginate. In LPC solution at 1 mg/mL, the complexation was at its maximum with sodium alginate solution (10 mg/mL) at an LPC:alginate ratio of 10:1 at a pH between 2.20 and 2.80, depending on the LPC type; the complex yield increased with the increase in LPC protein content. Under these conditions, the entrapment efficiency of folic acid was high (approximately 90%) for the RuBisCO-rich fractions; indeed, the computational protein–ligand docking predicted a strong binding affinity between folic acid and RuBisCO. The in vitro simulated GIT release study showed favorable release kinetics of folic acid, so leaf proteins, in combination with charged polysaccharides, may act as effective carriers of sensitive vitamins.

## Figures and Tables

**Table 1:** Chemical analysis of leaf protein concentrates.

Sample	Yield, % (g/100 g Dry Leaf)	Carbohydrates, % (g/100 g Powder)	Proteins (Kjeldahl), % (g/100 g Powder)	TPC * (mg GAE/g)
LPC_TA_	1.31 ± 0.07 ^b^**	3.70 ± 0.31 *^cd^*	83.23 ± 1.05 *^b^*	0.277 ± 0.002 *^a^*
LPC_AA_	0.92 ± 0.07 *^c^*	6.23 ± 0.42 *^c^*	74.82 ± 1.20 *^c^*	0.250 ± 0.051 *^b^*
LPC_40_	2.30 ± 0.12 *^a^*	5.20 ± 0.20 *^c^*	90.22 ± 1.55 *^a^*	0.180 ± 0.010 *^c^*
LPC_60_	1.14 ± 0.06 *^b^*	10.6 ± 0.20 *^b^*	80.28 ± 1.02 *^b^*	0.290 ± 0.010 *^a^*
LPC_80–100_	0.50 ± 0.04 *^d^*	21.3 ± 0.40 *^a^*	62.71 ± 1.64 *^d^*	0.241 ± 0.002 *^b^*

* Total phenolic content. ** Results are expressed as mean ± standard deviation (*n* = 2). Means with different letters in the same column are significantly different (*p* < 0.05).

**Table 2:** Influence of the LPC-alginate ratio on the complexes yield and the encapsulation efficiency of folic acid.

LPC:Alginate Ratio	2:1	5:1	10:1	
Sample	Cy %	Cy, %	Cy, %	EE, %
LPC_TA_	nd *	nd	67.9 ± 3.9 ^a^**	85.2 ± 3.8 *^a^*
LPC_AA_	nd	nd	71.0 ± 4.4 *^a^*	92.1 ± 4.6 *^a^*
LPC_40_	nd	nd	64.3 ± 3.6 *^a^*	88.3 ± 4.2 *^a^*
LPC_60_	nd	46.7 ± 2.5 *^b^*	51.2 ± 2.5 *^b^*	65.2 ± 2.1 *^b^*
LPC_80–100_	nd	nd	42.3 ± 3.2 *^b^*	46.4 ± 3.7 *^c^*

* Not determined; ** Results are expressed as mean ± standard deviation (*n* = 2). Means with different letters in the same column are significantly different (*p* < 0.05).

## Data Availability

The original contributions presented in the study are included in the article, further inquiries can be directed to the corresponding author.

## References

[1] Turgeon S.L., Laneuville S.I., Kasapis S., Norton I.T., Ubbink J.B. (2009). Protein +Polysaccharide Coacervates and Complexes: From Scientific Background to their Application as Functional Ingredients in Food Products. Modern Biopolymer Science.

[2] De Kruif C.G., Weinbreck F., De Vries R. (2004). Complex coacervation of proteins and anionic polysaccharides. Curr. Opin. Colloid Interface Sci..

[3] Eghbal N., Choudhary R. (2018). Complex coacervation: Encapsulation and controlled release of active agents in food systems. LWT.

[4] Devi N., Sarmah M., Khatun B., Maji T.K. (2017). Encapsulation of active ingredients in polysaccharide–protein complex coacervates. Adv. Colloid Interface Sci..

[5] Timilsena Y.P., Akanbi T.O., Khalid N., Adhikari B., Barrow C.J. (2019). Complex coacervation: Principles, mechanisms and applications in microencapsulation. Int. J. Biol. Macromol..

[6] Muhoza B., Qi B., Harindintwali J.D., Koko M.Y.F., Zhang S., Li Y. (2022). Combined plant protein modification and complex coacervation as a sustainable strategy to produce coacervates encapsulating bioactives. Food Hydrocoll..

[7] Nieuwland M., Geerdink P., Engelen-Smit N.P., Van Der Meer I.M., America A.H., Mes J.J., Mulder W.J. (2021). Isolation and gelling properties of duckweed protein concentrate. ACS Food Sci. Technol..

[8] Ishikawa C., Hatanaka T., Misoo S., Miyake C., Fukayama H. (2011). Functional incorporation of sorghum small subunit increases the catalytic turnover rate of Rubisco in transgenic rice. Plant Physiol..

[9] Shen Z.X., Li S.Y. (2023). Increasing the atom economy of glucose fermentation for bioethanol production in Rubisco-based engineered Escherichia coli. Bioresour. Technol. Rep..

[10] Martin A.H., Castellani O., de Jong G.A., Bovetto L., Schmitt C. (2019). Comparison of the functional properties of RuBisCO protein isolate extracted from sugar beet leaves with commercial whey protein and soy protein isolates. J. Sci. Food Agric..

[11] Tan Y., Lee P.W., Martens T.D., McClements D.J. (2022). Comparison of emulsifying properties of plant and animal proteins in oil-in-water emulsions: Whey, soy, and RuBisCo proteins. Food Biophys..

[12] Tenorio A.T., Gieteling J., De Jong G.A., Boom R.M., Van Der Goot A.J. (2016). Recovery of protein from green leaves: Overview of crucial steps for utilisation. Food Chem..

[13] Santamaría-Fernández M., Lübeck M. (2020). Production of leaf protein concentrates in green biorefineries as alternative feed for monogastric animals. Anim. Feed Sci. Technol..

[14] Kornet R., Roozalipour S.L., Venema P., van der Goot A.J., Meinders M.B., van der Linden E. (2022). Coacervation in pea protein solutions: The effect of pH, salt, and fractionation processing steps. Food Hydrocoll..

[15] Biswas S., Hecht A.L., Noble S.A., Huang Q., Gillilan R.E., Xu A.Y. (2023). Understanding the impacts of molecular and macromolecular crowding agents on protein–polymer complex coacervates. Biomacromolecules.

[16] Razzak M.A., Cho S.J. (2023). Physicochemical and functional properties of capsaicin loaded cricket protein isolate and alginate complexes. J. Colloid Interface Sci..

[17] Wingfield P. (1998). Protein precipitation using ammonium sulfate. Curr. Protocol. Protein Sci..

[18] Tola A.J., Missihoun T.D. (2023). Ammonium sulfate-based prefractionation improved proteome coverage and detection of carbonylated proteins in *Arabidopsis thaliana* leaf extract. Planta.

[19] Chapeau A.L., Bertrand N., Briard-Bion V., Hamon P., Poncelet D., Bouhallab S. (2017). Coacervates of whey proteins to protect and improve the oral delivery of a bioactive molecule. J. Funct. Foods.

[20] Yingleardrattanakul P., Thompson A.K., Taprap R., Pinsirodom P., Bindu C. (2024). Evaluation of the efficiency of various folic acid microencapsulation techniques for rice vermicelli (Khanom Jeen) fortification. Food Humanit..

[21] Ghosh S., Melton L.D., Kihara S., Xu A., Mata J., Whitten A., Rekas A., McGillivray D. (2024). Effect of Encapsulation and Freeze-Drying on the Structural Organisation of Β-Lactoglobulin-Pectin Complex Coacervates with Bioactive Molecules. https://www.researchgate.net/publication/384587790_Effect_of_Encapsulation_and_Freeze-Drying_on_the_Structural_Organisation_of_B-Lactoglobulin_-_Pectin_Complex_Coacervates_with_Bioactive_Molecules.

[22] Xie Y., Liu Q., Ge Y., Liu Y., Yang R. (2024). Formation and Applications of Typical Basic Protein-Based Heteroprotein Complex Coacervations. Foods.

[23] (2005). Animal Feeding Stuffs: Determination of Nitrogen Content and Calculation of Crude Protein Content—Part 1: Kjeldahl Method.

[24] DuBois M., Gilles K.A., Hamilton J.K., Rebers P.T., Smith F. (1956). Colorimetric method for determination of sugars and related substances. Anal. Chem..

[25] Skotti E., Anastasaki E., Kanellou G., Polissiou M., Tarantilis P.A. (2014). Total phenolic content, antioxidant activity and toxicity of aqueous extracts from selected Greek medicinal and aromatic plants. Ind. Crops Prod..

[26] Laemmli U. (1970). Cleavage of Structural Proteins during the Assembly of the Head of Bacteriophage T4. Nature.

[27] Liu Y., Yang X., Gan J., Chen S., Xiao Z.X., Cao Y. (2022). CB-Dock2: Improved protein–ligand blind docking by integrating cavity detection, docking and homologous template fitting. Nucleic Acids Res..

[28] Yang X., Liu Y., Gan J., Xiao Z.X., Cao Y. (2022). FitDock: Protein–ligand docking by template fitting. Brief. Bioinf..

[29] The UniProt Consortium (2018). UniProt: The universal protein knowledgebase. Nucleic Acids Res..

[30] Kim S., Chen J., Cheng T., Gindulyte A., He J., He S., Li Q., Shoemaker B.A., Thiessen P.A., Yu B. (2023). PubChem 2023 update. Nucleic Acids Res..

[31] Liu W., Ye A., Liu C., Liu W., Singh H. (2012). Structure and integrity of liposomes prepared from milk-or soybean-derived phospholipids during in vitro digestion. Food Res. Int..

[32] Pérez-Vila S., Fenelon M.A., O’Mahony J.A., Gómez-Mascaraque L.G. (2022). Extraction of plant protein from green leaves: Biomass composition and processing considerations. Food Hydrocoll..

[33] Everette J.D., Bryant Q.M., Green A.M., Abbey Y.A., Wangila G.W., Walker R.B. (2010). Thorough study of reactivity of various compound classes toward the Folin−Ciocalteu reagent. J. Agric. Food. Chem..

[34] Derardja A.E., Pretzler M., Kampatsikas I., Radovic M., Fabisikova A., Zehl M., Rompel A. (2022). Polyphenol oxidase and enzymatic browning in apricot (*Prunus armeniaca* L.): Effect on phenolic composition and deduction of main substrates. Curr. Res. Food Sci..

[35] Perović M.N., Jugović Z.D.K., Antov M.G. (2024). Heat-induced nanoparticles from pumpkin leaf protein for potential application as β-carotene carriers. Future Foods.

[36] Ghaly A.E., Alkoaik F.N. (2010). Extraction of protein from common plant leaves for use as human food. Am. J. Appl. Sci..

[37] Martin A.H., Nieuwland M., de Jong G.A. (2014). Characterization of heat-set gels from RuBisCO in comparison to those from other proteins. J. Agric. Food. Chem..

[38] Doubnerová V., Ryšlavá H. (2011). What can enzymes of C4 photosynthesis do for C3 plants under stress?. Plant Sci..

[39] Famuwagun A.A., Alashi A.M., Gbadamosi S.O., Taiwo K.A., Oyedele D.J., Adebooye O.C., Aluko R.E. (2020). Comparative study of the structural and functional properties of protein isolates prepared from edible vegetable leaves. Int. J. Food Prop..

[40] Valente A.I., Ferreira A.M., Almeida M.R., Mohamadou A., Freire M.G., Tavares A.P. (2021). Efficient Extraction of the RuBisCO Enzyme from Spinach Leaves Using Aqueous Solutions of Biocompatible Ionic Liquids. Sustain. Chem..

[41] Yücetepe A., Yavuz-Düzgün M., Şensu E., Bildik F., Demircan E., Özçelik B. (2021). The impact of pH and biopolymer ratio on the complex coacervation of Spirulina platensis protein concentrate with chitosan. J. Food Sci. Technol..

[42] Jones O.G., Lesmes U., Dubin P., McClements D.J. (2010). Effect of polysaccharide charge on formation and properties of biopolymer nanoparticles created by heat treatment of β-lactoglobulin–pectin complexes. Food Hydrocoll..

[43] Zheng J., Van der Meeren P., Sun W. (2024). New insights into protein–polysaccharide complex coacervation: Dynamics, molecular parameters, and applications. Aggregate.

[44] Wu Z., Li X., Hou C., Qian Y. (2010). Solubility of folic acid in water at pH values between 0 and 7 at temperatures (298.15, 303.15, and 313.15) K. J. Chem. Eng. Data..

[45] Saravanan M., Rao K.P. (2010). Pectin–gelatin and alginate–gelatin complex coacervation for controlled drug delivery: Influence of anionic polysaccharides and drugs being encapsulated on physicochemical properties of microcapsules. Carbohydr. Polym..

[46] Liang L., Subirade M. (2010). β-Lactoglobulin/folic acid complexes: Formation, characterization, and biological implication. J. Phys. Chem. B.

[47] Zhang J., Liu X., Subirade M., Zhou P., Liang L. (2014). A study of multi-ligand beta-lactoglobulin complex formation. Food Chem..

[48] Tavares G.M., Croguennec T., Lê S., Lerideau O., Hamon P., Carvalho A.F., Bouhallab S. (2015). Binding of folic acid induces specific self-aggregation of lactoferrin: Thermodynamic characterization. Langmuir.

[49] Chapeau A.L., Hamon P., Rousseau F., Croguennec T., Poncelet D., Bouhallab S. (2017). Scale-up production of vitamin loaded heteroprotein coacervates and their protective property. J. Food Eng..

[50] Ding X., Yao P. (2013). Soy protein/soy polysaccharide complex nanogels: Folic acid loading, protection, and controlled delivery. Langmuir.

[51] Zhang S., Zhang Z., Vardhanabhuti B. (2014). Effect of charge density of polysaccharides on self-assembled intragastric gelation of whey protein/polysaccharide under simulated gastric conditions. Food Funct..

[52] Strugala V., Kennington E.J., Campbell R.J., Skjåk-Bræk G., Dettmar P.W. (2005). Inhibition of pepsin activity by alginates in vitro and the effect of epimerization. Int. J. Pharm..

[53] Chater P.I., Wilcox M.D., Brownlee I.A., Pearson J.P. (2015). Alginate as a protease inhibitor in vitro and in a model gut system; selective inhibition of pepsin but not trypsin. Carbohydr. Polym..

[54] Wang B., Blanch E., Barrow C.J., Adhikari B. (2017). Preparation and study of digestion behavior of lactoferrin-sodium alginate complex coacervates. J. Funct. Foods.

[55] Bernstein L.H., Gutstein S., Weiner S., Efron G. (1970). The absorption and malabsorption of folic acid and its polyglutamates. Am. J. Med..

